# Germline variants at *SOHLH2* influence multiple myeloma risk

**DOI:** 10.1038/s41408-021-00468-6

**Published:** 2021-04-19

**Authors:** Laura Duran-Lozano, Gudmar Thorleifsson, Aitzkoa Lopez de Lapuente Portilla, Abhishek Niroula, Molly Went, Malte Thodberg, Maroulio Pertesi, Ram Ajore, Caterina Cafaro, Pall I. Olason, Lilja Stefansdottir, G. Bragi Walters, Gisli H. Halldorsson, Ingemar Turesson, Martin F. Kaiser, Niels Weinhold, Niels Abildgaard, Niels Frost Andersen, Ulf-Henrik Mellqvist, Anders Waage, Annette Juul-Vangsted, Unnur Thorsteinsdottir, Markus Hansson, Richard Houlston, Thorunn Rafnar, Kari Stefansson, Björn Nilsson

**Affiliations:** 1 Hematology and Transfusion Medicine, Department of Laboratory Medicine, 221 84 Lund, Sweden; 2 deCODE genetics, Sturlugata 8, IS-101 Reykjavik, Iceland; 3 Broad Institute, 415 Main Street, Cambridge, MA 02124 USA; 4 Division of Genetics and Epidemiology, The Institute of Cancer Research, 123 Old Brompton Road, London, SW7 3RP UK; 5 Hematology Clinic, Lund University Hospital, 221 85 Lund, Sweden; 6 Department of Internal Medicine V, University Hospital of Heidelberg, 69120 Heidelberg, Germany; 7 Hematology Research Unit, Department of Clinical Research, University of Southern Denmark and Department of Hematology, Odense University Hospital, Odense, Denmark; 8 Department of Haematology, Aarhus University Hospital, 8200 Aarhus N, Denmark; 9 Södra Älvsborgs Sjukhus Borås, Borås, Sweden; 10 Institute of Clinical and Molecular Medicine, Norwegian University of Science and Technology, Department of Hematology, and Biobank1, St Olavs hospital, Trondheim, Norway; 11 Department of Haematology, University Hospital of Copenhagen at Rigshospitalet, Blegdamsvej 9, DK-2100 Copenhagen, Denmark; 12 Faculty of Medicine, University of Iceland, Reykjavik, Iceland

**Keywords:** Genetics research, Disease genetics

## Abstract

Multiple myeloma (MM) is caused by the uncontrolled, clonal expansion of plasma cells. While there is epidemiological evidence for inherited susceptibility, the molecular basis remains incompletely understood. We report a genome-wide association study totalling 5,320 cases and 422,289 controls from four Nordic populations, and find a novel MM risk variant at *SOHLH2* at 13q13.3 (risk allele frequency = 3.5%; odds ratio = 1.38; *P* = 2.2 × 10^−14^). This gene encodes a transcription factor involved in gametogenesis that is normally only weakly expressed in plasma cells. The association is represented by 14 variants in linkage disequilibrium. Among these, rs75712673 maps to a genomic region with open chromatin in plasma cells, and upregulates *SOHLH2* in this cell type. Moreover, rs75712673 influences transcriptional activity in luciferase assays, and shows a chromatin looping interaction with the *SOHLH2* promoter. Our work provides novel insight into MM susceptibility.

## Introduction

Multiple myeloma (MM) is characterized by an uncontrolled, clonal expansion of plasma cells in the bone marrow, producing a monoclonal immunoglobulin (“M protein”). Clinically, MM is complicated by bone marrow and kidney failure, hypercalcemia, and lytic bone lesions^1^. MM is preceded by monoclonal gammopathy of undetermined significance (MGUS)^2,3^, detectable in 3% of individuals older than 50 years^4^, which progresses to MM at an annual rate of about 1%^5^.

Epidemiological studies have shown that first-degree relatives of patients with MM have 2–4 times higher risk of MM and MGUS^6–8^, as well as an increased risk of chronic lymphocytic leukemia, lymphomas, and certain solid tumours^9–11^. Genome-wide association studies (GWAS) have identified inherited sequence variants at 24 loci that influence MM risk and sequencing studies of familial cases have implicated candidate genes where rare MM-predisposing variants might reside^12^. Still, identified loci explain less than 20% of the estimated heritability^13^.

To advance our understanding of MM genetics, we carried out a GWAS based on cases and controls from four Nordic populations, with follow-up in additional data sets of European ancestry. We detect a novel MM risk locus at 13q13.3, spanning the *SOHLH2* gene. Dissecting the functional architecture, we identify a putative causal variant within the linkage disequilibrium (LD) block that likely acts by upregulating *SOHLH2* in plasma cells.

## Methods

### Sample sets

We carried out a GWAS totalling 5,320 MM and non-IgM MGUS cases and 422,293 controls recruited from Denmark, Iceland, Norway and Sweden (Tables 1 and 2); Swedish series 2,338 MM cases from the Swedish National MM Biobank (Skåne University Hospital, Lund) and 11,971 Swedish controls (random blood donors and primary care patients) genotyped within an ongoing GWAS on blood cell traits (Lopez de Lapuente Portilla et al., ongoing). Danish series 940 MM cases from Rigshospitalet in Copenhagen, Odense University Hospital, and Aarhus University Hospital and 91,744 controls from the Danish Blood Donor Study^14^; Norwegian series 500 MM cases from the Norwegian MM Biobank (St. Olavs Hospital-Trondheim University Hospital) and 4,696 blood donor controls (Oslo University Hospital, Ullevål Hospital); Icelandic series 598 MM cases, 944 non-IgM MGUS cases and 313,882 controls from the deCODE Genetics database^15^. Information on cases with MM and non-IgM MGUS was obtained from Landspitali – The University Hospital of Iceland and the Icelandic Cancer Registry^16^. The MGUS cases are individuals with MGUS who have not yet progressed to MM according to the Icelandic Cancer Registry.

**Table 1:** Association data for the 13q13 association signal.

Population	MM + MGUS					MM				
	Cases	Controls	OR	*P*- value	*P*_het_	Cases	Controls	OR	*P*-value	*P*_het_
*Discovery samples, rs200203825*										
Sweden	2,338	11,971	1.35	1.14 × 10^−3^		2,338	11,967	1.35	1.14 × 10^−3^	
Iceland	1,542	313,882	1.40	1.1 × 10^−4^		598	237,480	1.15	3.64 × 10^−1^	
Denmark	940	91,744	1.51	8.61 × 10^−5^		940	91,744	1.51	8.61 × 10^−5^	
Norway	500	4,696	1.16	4.16 × 10^−1^		500	4,696	1.16	4.16 × 10^−1^	
Combined discovery	5,320	422,293	1.39	2.65 × 10^−10^	0.63	4,376	345,887	1.34	7.37 × 10^−7^	0.39
*Discovery samples, rs75712673*										
Sweden	2,338	11,971	1.32	2.79 × 10^−3^		2,338	11,967	1.32	2.79 × 10^−3^	
Iceland	1,542	313,882	1.37	3.79 × 10^−4^		598	237,480	1.20	2.14 × 10^−1^	
Denmark	940	91,744	1.52	6.05 × 10^−5^		940	91,744	1.52	6.05 × 10^−5^	
Norway	500	4,696	1.17	3.92 × 10^−1^		500	4,696	1.17	3.92 × 10^−1^	
Combined discovery	5,320	422,293	1.37	1.55 × 10^−9^	0.60	4,376	345,887	1.34	6.58 × 10^−7^	0.46
*Follow-up samples*										
Sweden^a^	473	3,430	1.26	1.63 × 10^−1^		473	3,430	1.26	1.63 × 10^−1^	
United Kingdom^b^	2,282	5,198	1.26	9.35 × 10^−3^		2,282	5,198	1.26	9.35 × 10^−3^	
USA^b^	780	1,857	1.28	1.03 × 10^−1^		780	1,857	1.28	1.03 × 10^−1^	
Germany^b^	1,508	2,107	1.44	1.47 × 10^−2^		1,508	2,107	1.44	1.47 × 10^−2^	
Netherlands^b^	555	2,669	1.49	1.26 × 10^−2^		555	2,669	1.49	1.26 × 10^−2^	
Combined follow-up	5,598	15,261	1.32	2.6 × 10^−6^	0.86	5,598	15,261	1.32	2.6 × 10^−6^	0.86
Combined (discovery + follow-up)	10,918	437,554	1.35	2.2 × 10^−14^	0.64	9,974	361,148	1.33	7.9 × 10^−12^	0.86

^a^proxy SNP rs78351393 genotype.

^b^rs75712673 genotype.

**Table 2:** Characteristics of study populations.

	Cases			Controls	
	Individuals	Male (%)	Mean age diagnosis	Individuals	Male (%)
*Discovery samples*					
Sweden (age data for 1,855)	2,338	56.8	69.1	11,971	47.8
Iceland, MM + MGUS (age for 137/944)	1,542	50.7	67.0	313,882	51.2
Iceland, MM (age for 457/598)	598	52.8	70.0	237,480	51.1
Denmark	940	59.4	n/a	91,744	51.0
Norway	500	60.0	n/a	4,696	53.9
*Follow-up samples*					
Sweden	473	62.2	69.4	3,430	45.7

For follow-up, we analysed: (1) an additional 473 MM cases from the Swedish National MM Biobank and 3,430 Swedish controls (a second cohort of random blood donors and primary care patients) from an ongoing GWAS on blood cell traits (Lopez de Lapuente Portilla et al., ongoing); (2) pre-existing GWAS data from the United Kingdom, Germany, USA and the Netherlands^13^ (Table 1).

All samples were collected with informed consent and ethical approval (Lund University Ethical Review Board, 2013/54; Rigshospitalet Ethical Committee no. 69466; Icelandic National Bioethics Committee ref. 17–143; Regional Committee for Medical and Health Research Ethics, Trondheim 2014/97; Regional Committee for Medical and Health Research Ethics, Oslo), and in accordance with the principles of the Declaration of Helsinki.

### Genotyping and imputation

Swedish, Danish and Norwegian samples were genotyped with Illumina single-nucleotide polymorphism microarrays and phased together with 442,737 samples from North-Western Europe using Eagle2^17^. Samples and variants with <98% yield were excluded. We used the same methods as used for the Icelandic data^15,18^ to create a haplotype reference panel by phasing the whole-genome sequence (WGS) genotypes for 15,575 individuals from North-Western Europe, including 3,012 Swedish, 8,429 Danish and 2,550 Norwegian samples, together with the phased microarray data, and to impute the genotypes from the haplotype reference panel into the phased microarray data.

Sample preparation and WGS of 49,962 Icelanders are previously reported^15,19^. Briefly, 37.6 million sequence variants were identified by WGS in 49,962 Icelanders using Illumina technology to a mean depth of at least 18×. SNPs and indels were identified and their genotypes were called jointly using Graphtyper^20^. In addition, over 165,000 Icelanders, including all those with WGS data, have been microarray-genotyped and long-range phased^18^, improving genotype calls using information based on haplotype sharing. The genotypes of the high-quality sequence variants were imputed into the microarray-typed Icelanders^21^. To increase the sample size and power to detect associations, the sequence variants were also imputed into relatives of the microarray-typed using genealogic information. All tested variants had imputation information > 0.8. Variants were mapped to hg38 and matched on position and alleles to harmonize the four data sets. rs145374408, rs78351393 and rs17202418 were genotyped in the Swedish follow-up sample set.

### Ancestry analysis

Genetic ancestry analysis was done in two stages for the Danish, Swedish and Norwegian sample sets separately. Firstly, ADMIXTURE v1.23^22^ was run in supervised mode with 1000 Genomes populations CEU, CHB, and YRI^23^ as training samples and Danish, Swedish or Norwegian individuals as test samples. Input data for ADMIXTURE had long-range LD regions removed^24^ and was then LD-pruned with PLINK v.190b3a^25^ using the–indep-pairwise 200 25 0.3 option. Samples with <0.9 CEU ancestry were excluded. Secondly, remaining samples were projected onto a principal component analysis (PCA), calculated with an in-house European reference panel to calculate the 20 first principal components for each population. UMAP^26^ was used to reduce the coordinates of test samples on 20 principal components to two dimensions. Additional European samples not in the original reference set were also projected onto the PCA and UMAP components to identify ancestries represented in the clusters, and samples with Swedish, Danish and Norwegian ancestries were identified.

### Association testing

We performed logistic regression in the Icelandic, Swedish, Danish and Norwegian data set separately to test for association between MM and genotypes using deCODE software^15^. In the Danish, Swedish and Norwegian association analysis, we adjusted for gender, whether the individual had been microarray-typed and/or sequenced, and the first 20 principal components. In the Icelandic association analysis, we adjusted for gender, county-of-origin, current age or age at death, blood sample availability for the individual, and an indicator function for the overlap of the lifetime of the individual with the time span of phenotype collection. We used LD score regression to account for distribution inflation due to cryptic relatedness and population stratification^27^.

For the meta-analysis, we used a fixed-effects inverse variance method^28^. Of note, using a large number of controls, primarily in the Icelandic and Danish data sets, will not bias the results for individual data sets as it only provides more accurate estimate of the allelic frequency in the control group and hence increases power. The inverse variance method used to combine effect size estimates, in essence, weights effects by sample size through the use of corresponding standard errors. This meta-analysis method is well recognized and will not bias results when the ratio of cases to controls is unequal^29^.

Genome-wide significance was determined using class-based Bonferroni significance thresholds for about 33 million variants. Sequence variants were split into five classes based on their genome annotation, and the significance threshold for each class was based on the number of variants in that class^30^. The adjusted significance thresholds used are 2.49 × 10^−7^ for variants with high impact (including stop-gained and stop-loss, frameshift, splice acceptor or donor and initiator codon variants), 4.97 × 10^−8^ for variants with moderate impacts (including missense, splice-region variants, inframe deletions and insertions), 4.52 × 10^−9^ for low-impact variants (including synonymous, 3′ and 5′ UTR, and upstream and downstream variants), 2.26 × 10^−9^ for deep intronic and intergenic variants in DNase I hypersensitivity sites and 7.53 × 10^−10^ for all other variants, including those in intergenic regions.

### eQTL analysis

To identify expression quantitative locus (eQTL) effects in plasma cells, we used previously published RNA-seq data for CD138^+^ immunomagnetic bead-enriched MM plasma cells^31^. To test for association, we used linear regression with and without 10 principal components as covariates. To identify eQTLs in blood, we used whole-blood RNA-seq from 13,127 Icelanders^32^. Gene expression was quantified based on transcript abundances estimates using Kallisto^33^. Association between sequence variants and gene expression was calculated using generalized linear regression^34^. The additive genetic effect was assumed and quantile-normalized gene expression estimates were calculated while adjusting for sequencing artefacts, demography variables, and hidden factors^35^. Finally, as a complement to the Icelandic data, we used data from the eQTLGen database (www.eqtlgen.org)^36^.

### ATAC-seq data for blood cell populations

Sequencing reads for published ATAC-seq libraries from sorted hematopoietic cell types were downloaded from https://atac-blood-hg38.s3.amazonaws.com/hg38/ using the rtracklayer R package^37^, and processed using hg38 as a reference genome.

### Chromatin immunoprecipitation sequencing

As a complementary approach to identify variants located in genomic regions with regulatory activity in plasma cells, we analyzed previously published^31^ chromatin immunoprecipitation sequencing (ChIP-seq) data for the H3K4me3 histone modification^38^. Briefly, L363 cells (DSMZ) were cross-linked with 1% paraformaldehyde (ThermoFisher, #28908). DNA was sonicated into 200–400 bp fragments (Bioruptor Pico Sonication System, Diagenode, Belgium). For pull-down, we used 1–10 μg of H3K4me3 antibody (Millipore, #04-745). Fragments were de-cross-linked and purified (Zymogen, #D5205). ChIP-seq libraries were prepared using the ThruPLEX DNA-seq Kit (Rubicon Genomics, #R400406) and sequenced on Illumina HiSeq 2500 sequencer (paired-end; 2 × 125 cycles). De-multiplexing and generation of FASTQ files was performed using bcl2fastq v.1.8. FastQC (v0.11.5)^39^ was used to assess read quality low-quality bases were removed using Trimmomatic (v.0.36)^40,41^ prior to alignment. using Bowtie2 (v.2.3.0)^41^. Coverage in 50 bp over the *SOHL2* region was calculated with the GenomicAlignments and GenomicRanges R-packages^42^ (coverage and binnedAverage functions) and scaled to Counts-per-million (CPM) relative to the total number of reads per library.

### Luciferase assays

Luciferase constructs representing the reference and alternative allele of rs75712673 were made by cloning 120-bp genomic sequences (Integrated DNA Technologies) centered on the variant into the pGL3-basic vector. Using electroporation (Neon Transfection system; Life technologies, USA) constructs were co-transfected with renilla plasmid into L363 and OPM2 cells (DSMZ). Twenty hours after electroporation, luciferase and renilla activity was measured using DualGlo Luciferase (Promega no. E1960) on a GLOMAX 20/20 Luminometer (Promega, USA). Based on luciferase/renilla readings, we calculated log_2_ scores for each variant.

### Transcription factor motif analysis

To identify differentially binding transcription factors, we used the PERFECTOS-APE tool (http://opera.autosome.ru/perfectosape) with the HOCOMOCO-10, JASPAR, HT-SELEX, SwissRegulon and HOMER motif databases.

### Figure generation

Region plots were generated using tidyGenomeBrowser (https://github.com/MalteThodberg/tidyGenomeBrowser). Transcript models were obtained via TxDb.Hsapiens.UCSC.hg38.knownGene and org.Hs.eg.db packages^43^ and collapsed to meta gene models with the exonsBy-function.

## Results

### Identification of a novel MM risk locus at 13q13

To find new MM risk loci, we carried out a GWAS based on four case-control data sets from Iceland, Denmark, Norway and Sweden totalling 5,320 MM patients and 422,289 controls (Table 1). We performed association testing in the four data sets separately, and combined the resulting statistics for 33 million variants that passed quality filtering. Two versions of the Icelandic case-control data were used for meta-analysis: one with MM patients only, and one that was expanded with non-IgM MGUS patients to increase power (Table 1). The latter is motivated because MM evolves from MGUS, relatives of MGUS patients have increased MM risk, and several studies support pleiotropy between MM and MGUS^44^.

Our analysis identified genome-wide significant association signals at 10 loci, and all previously reported MM lead variants were nominally significant with effects in the same direction as in the discovery studies (Supplementary Table 1). Nine of the genome-wide significant signals correspond to signals correspond to known MM risk loci. In addition, a previously unreported low-frequency variant at 13q13.3 (lead variant rs200203825; RAF ~ 3,5%; combined *P* = 2.65 × 10^−10^ with Icelandic MGUS cases; Table 1) showed significant association. This signal is represented by a haplotype of 14 non-coding variants in high LD (*r*^*2*^ > 0.8) spanning the *SOHLH2* gene (spermatogenesis and oogenesis specific basic helix-loop-helix 2; Supplementary Table 2). The detected variants showed comparable effects in the same direction in all four discovery sets (Table 1). The conditional analysis did not reveal any additional independent signals. For follow-up, we genotyped an additional 473 MM cases and 3,430 controls from Sweden, and also looked for association in published MM association data sets from the United Kingdom, Germany, Holland and USA (Table 1). The association with MM was nominally significant in three of the six follow-up data sets, including in the two largest series from the UK and Germany. For all the series the effects were in the same direction as in our discovery GWAS.

### Functional annotation of the 13q13.3 association

Because non-coding variants generally act by altering the regulation of gene expression, we sought to identify candidate causal variants responsible for the 13q13.3 association based on epigenetic features associated with regulatory activity.

**Fig. 1:**
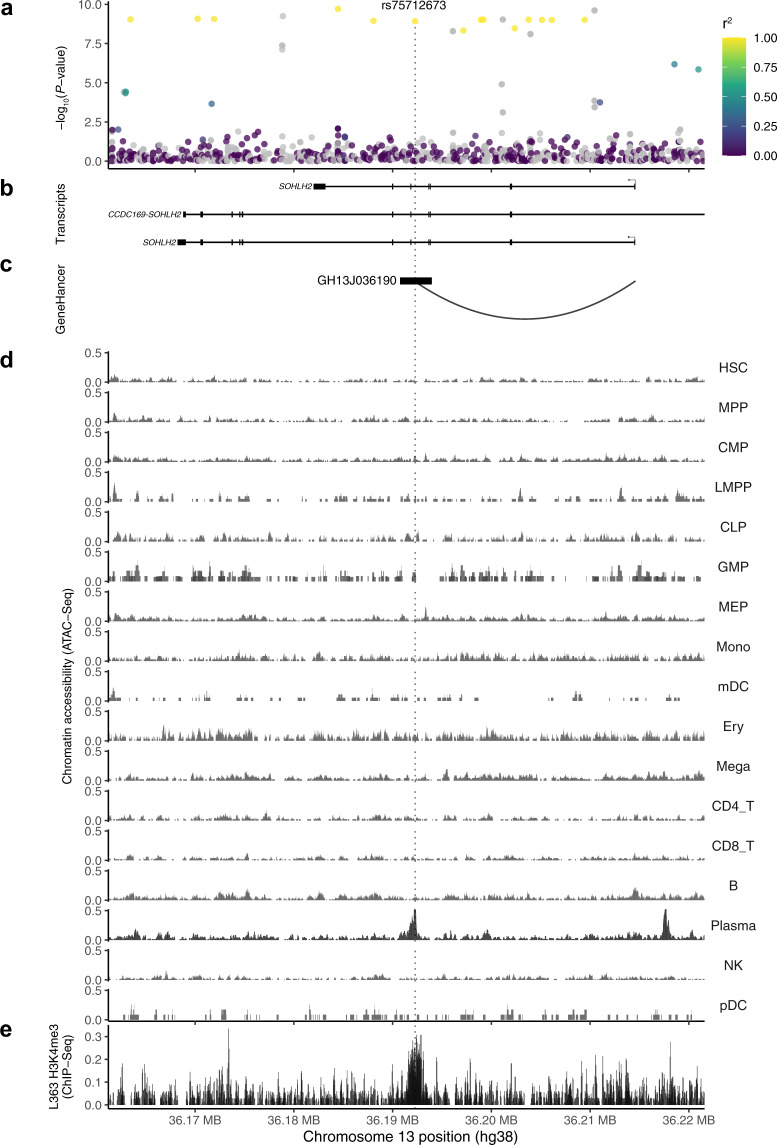
Region plots of the 13q13.3 association. **a** −log_10_(*P*) for association in the meta-analysis of the four Nordic discovery sets (*y*-axis). The colour reflects the extent of LD with the 13q13.3 lead variant rs200203825. **b** Genes mapping to the region of association, based on NCBI build 38 of the human genome. **c** Chromatin looping interaction between rs75712673 and the *SOHLH2* promoter, detected in promoter-capture HiC in transformed fibroblasts, data from GeneHancer **d** ATAC-seq chromatin accessibility across primary blood cell types; rs75712673 maps to a region that is selectively open in plasma cells. **e** Chromatin immunoprecipitation and sequencing (ChIP-seq) data for H3K4me3 histone mark in L363 MM plasma cell line.

Firstly, ATAC-seq data from 17 blood cell subpopulations showed that rs75712673 (*r*^*2*^ = 0.985 with rs200203825 in Swedes) maps to a genomic region selectively open in plasma cells (Fig. 1d). Secondly, consistent with this, chromatin immunoprecipitation and sequencing (ChIP-seq) data for the MM plasma cell line L363, showed that rs75712673 maps to a H3K4me3 histone mark (Fig. 1e). Collectively, these data are consistent with the 13q13.3 association affecting regulatory activity at rs75712673 in plasma cells.

**Fig. 2:**
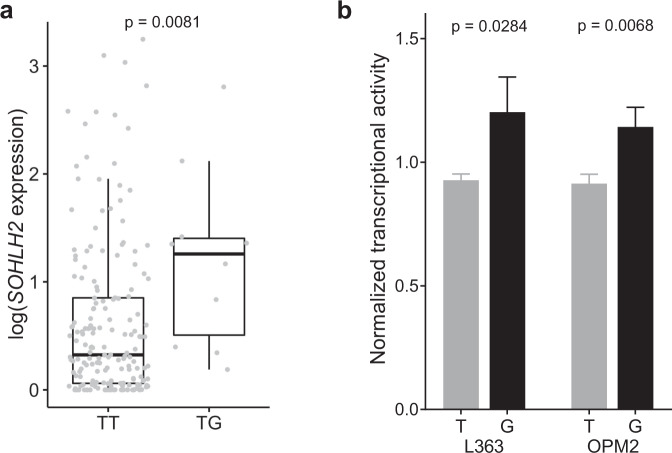
Candidate variant rs75712673 eQTL effect on *SOHLH2*. **a** Correlation of genotype and log transformed RNA-seq data from CD138 + plasma cell isolated from the bone marrow of 188 MM patients (Wilcoxon *p* = 0.0081). **b** Luciferase activity of rs75712673 reference and alternative alleles in two MM cell lines, L363 (*t*-test *p* = 0.0284) and OPM2 (*t*-test *p* = 0.0068) shows that regulation of *SOHLH2* expression varies in the two alleles of rs75712673. Error bars indicate standard deviation.

To identify a putative target gene, we carried out eQTL analysis using RNA-seq data for CD138^+^ plasma cell isolated from the bone marrow of 188 MM patients^31^ (Fig. 2a). We identified an association between rs75712673 and *SOHLH2* expression (Wilcoxon rank sum test *P* = 0.0081 for heterozygotes *versus* major allele homozygotes without principal components; *P* = 0.043 with 10 expression and 10 genotype principal component covariates). By contrast, we did not detect a plasma cell-specific eQTL for any of the nearby genes *SPG20*, *DLCK1*, *CCDC169*, or *CCNA1*, nor a *SOHLH2* eQTL in peripheral blood. *SOHLH2* is normally expressed in hematopoietic stem and progenitor cells, with its expression in B-cells and plasma cells being low (Supplementary Fig. 1).

Further, consistent with the eQTL, luciferase analysis of rs75712673 in MM plasma cell lines L363 and OPM2 showed higher luciferase activity for the rs75712673-G risk allele relative to the reference allele (Fig. 2b). Computational motif analysis predicted that rs75712673-G alters multiple transcription factor motifs, including multiple members of the FOX and SOX families, IKZF2, and EN1 (Supplementary Table 3). Finally, using GeneHancer^45^ promoter-capture HiC data, we detected a chromatin looping interaction between rs75712673 and the *SOHLH2* promoter (Fig. 1c).

## Discussion

In conclusion, we have identified a new genetic association for MM at *SOHLH2*, increasing the number of risk loci to 25. This locus was not significant in our recent six-center meta-analysis totalling 9,974 cases. The likely reason was that the Went et al. study combined data from different geographic populations, and, moreover, that the imputation was done using non-population-matched reference genomes. By contrast, the present study was done in homogenous populations of Nordic ancestry and the imputation was done using population-matched reference genomes, increasing the power to detect low-frequency variants such as the one at *SOHLH2*.

Interestingly, *SOHLH2* encodes a transcription factor with a basic helix-loop-helix domain that has previously been implicated in spermatogenesis^46^ and development of breast and ovarian cancer^47^, but is normally expressed only at a low lever in plasma cells. To explore the mechanism underlying the *SOHLH2* association, we functionally fine-mapped the 13q13.3 signal, and identified rs75712673 as a likely causal variant that upregulates *SOHLH2* in plasma cells. Our findings provide novel insight into the molecular basis of inherited MM susceptibility.

## Supplementary information

Supplementary Figure 1

Supplementary Table 1

Supplementary Table 2

Supplementary Table 3
