# Author Correction: Germline variants at *SOHLH2* influence multiple myeloma risk

**DOI:** 10.1038/s41408-021-00575-4

**Published:** 2021-11-16

**Authors:** Laura Duran-Lozano, Gudmar Thorleifsson, Aitzkoa Lopez de Lapuente Portilla, Abhishek Niroula, Molly Went, Malte Thodberg, Maroulio Pertesi, Ram Ajore, Caterina Cafaro, Pall I. Olason, Lilja Stefansdottir, G. Bragi Walters, Gisli H. Halldorsson, Ingemar Turesson, Martin F. Kaiser, Niels Weinhold, Niels Abildgaard, Niels Frost Andersen, Ulf-Henrik Mellqvist, Anders Waage, Annette Juul-Vangsted, Unnur Thorsteinsdottir, Markus Hansson, Richard Houlston, Thorunn Rafnar, Kari Stefansson, Björn Nilsson

**Affiliations:** 1 Hematology and Transfusion Medicine, Department of Laboratory Medicine, 221 84 Lund, Sweden; 2 deCODE genetics, Sturlugata 8, IS-101 Reykjavik, Iceland; 3 Broad Institute, 415 Main Street, Cambridge, MA 02124 USA; 4 Division of Genetics and Epidemiology, The Institute of Cancer Research, 123 Old Brompton Road, London, SW7 3RP UK; 5 Hematology Clinic, Lund University Hospital, 221 85 Lund, Sweden; 6 Department of Internal Medicine V, University Hospital of Heidelberg, 69120 Heidelberg, Germany; 7 Hematology Research Unit, Department of Clinical Research, University of Southern Denmark and Department of Hematology, Odense University Hospital, Odense, Denmark; 8 Department of Haematology, Aarhus University Hospital, 8200 Aarhus N, Denmark; 9 Södra Älvsborgs Sjukhus Borås, Borås, Sweden; 10 Institute of Clinical and Molecular Medicine, Norwegian University of Science and Technology, Department of Hematology, and Biobank1, St Olavs hospital, Trondheim, Norway; 11 Department of Haematology, University Hospital of Copenhagen at Rigshospitalet, Blegdamsvej 9, DK-2100 Copenhagen, Denmark; 12 Faculty of Medicine, University of Iceland, Reykjavik, Iceland

**Keywords:** Genetics research, Disease genetics

Correction to: *Blood Cancer Journal* 10.1038/s41408-021-00468-6, published online 19 April 2021

After the publication of the original article, the author list was updated as given here:

Laura Duran-Lozano^1^, Gudmar Thorleifsson^2^, Aitzkoa Lopez de Lapuente Portilla^1^, Abhishek Niroula^1,3^, Molly Went^4^, Malte Thodberg^1^, Maroulio Pertesi^1^, Ram Ajore^1^, Caterina Cafaro^1^, Pall I. Olason^2^, Lilja Stefansdottir^2^, G. Bragi Walters^2^, Gisli H. Halldorsson^2^, Ingemar Turesson^5^, Martin F. Kaiser^4^, Asta Försti^6^, Hartmut Goldschmidt^7^, Kari Hemminki^6^, Niels Weinhold^8^, Niels Abildgaard^9^, Niels Frost Andersen^10^, Ulf-Henrik Mellqvist^11^, Anders Waage^12^, Annette Juul-Vangsted^13^, Unnur Thorsteinsdottir^2,14^, Markus Hansson^1,5^, Richard Houlston^4^, Thorunn Rafnar^2^, Kari Stefansson^2,14^, Björn Nilsson^1,3^

^1^Hematology and Transfusion Medicine, Department of Laboratory Medicine, 221 84 Lund, Sweden. ^2^deCODE genetics, Sturlugata 8, IS-101 Reykjavik, Iceland. ^3^Broad Institute, 415 Main Street, Cambridge MA 02124, USA. ^4^Division of Genetics and Epidemiology, The Institute of Cancer Research, 123 Old Brompton Road, London SW7 3RP, United Kingdom. ^5^Hematology Clinic, Lund University Hospital, 221 85 Lund, Sweden. ^6^German Cancer Research Center (DKFZ), Im Neuenheimer Feld 580, D-69120, Heidelberg, Germany. ^7^Department of Internal Medicine V, University of Heidelberg, Heidelberg, Germany, ^8^Department of Internal Medicine V, University Hospital of Heidelberg, 69120 Heidelberg, Germany. ^9^Hematology Research Unit, Department of Clinical Research, University of Southern Denmark and Department of Hematology, Odense University Hospital, Denmark. ^10^Department of Haematology, Aarhus University Hospital, 8200 Aarhus N, Denmark. ^11^Södra Älvsborgs Sjukhus Borås, Sweden. ^12^Institute of Clinical and Molecular Medicine, Norwegian University of Science and Technology, Department of Hematology, and Biobank1, St Olavs hospital, Trondheim, Norway. ^13^Department of Haematology, University Hospital of Copenhagen at Rigshospitalet, Blegdamsvej 9, DK-2100 Copenhagen, Denmark.^14^Faculty of Medicine, University of Iceland, Reykjavik, Iceland.

