# Supplementary material for: Germline variants at SOHLH2 influence multiple myeloma risk
Source: Blood Cancer J. 2021 Apr 19;11(4):76. doi: 10.1038/s41408-021-00468-6 (PMC8055668; doi:10.1038/s41408-021-00468-6)

Supplementary Figure 1

Expression of *SOHLH2* across human blood cell types. Data from **(a)** the differentiation map (D-MAP) and **(b)** the HemaExplorer datasets, both visualized using the Bloodspot portal (www.bloodspot.eu).

a

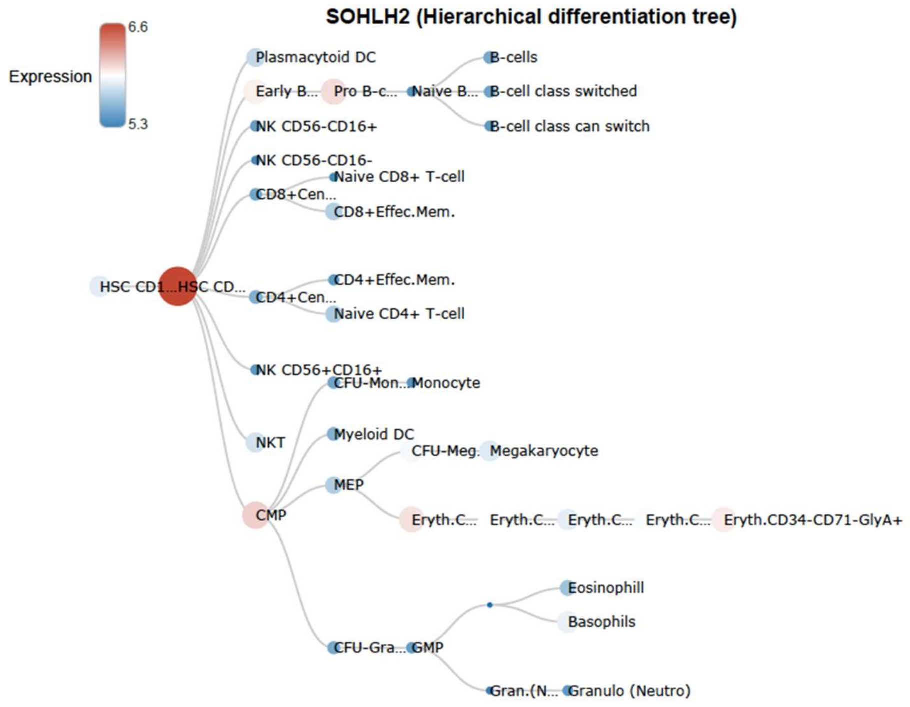

b

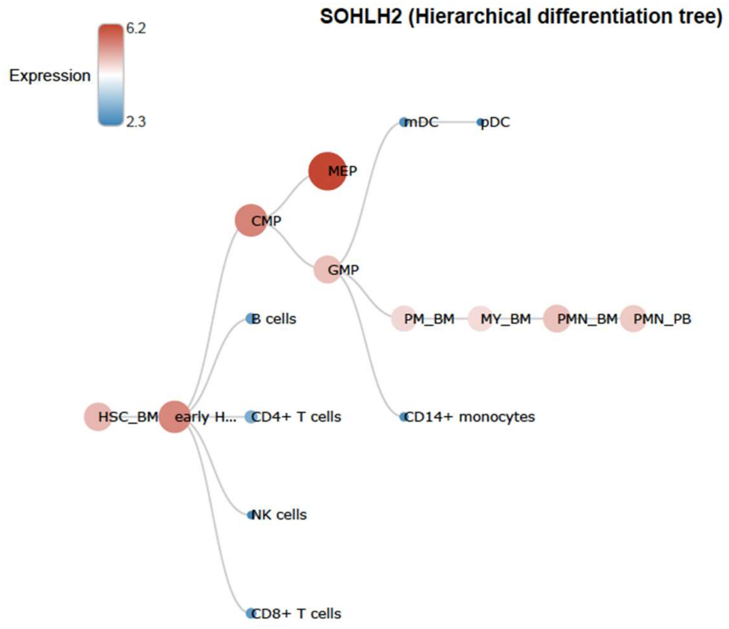

Supplement: Supplementary file 1 — Supplementary Figure 1 [file 41408_2021_468_MOESM1_ESM.pdf]
