# Supplementary material for: Germline variants at SOHLH2 influence multiple myeloma risk
Source: Blood Cancer J. 2021 Apr 19;11(4):76. doi: 10.1038/s41408-021-00468-6 (PMC8055668; doi:10.1038/s41408-021-00468-6)
Supplement: Supplementary file 2 — Supplementary Table 1 [file 41408_2021_468_MOESM2_ESM.pdf]

Supplementary Table 1

Association data for MM risk variants identified in previous, major genome-wide association studies.
RAF: risk allele frequency, extracted from Ensembl (1000 genomes Phase 3, EUR). *major alleles underlined

					Discovery study			Present study						
Candidate gene	locus	rsID	Risk allele*	RAF	Ref.	OR	*P* - value	Sweden freq	Iceland freq	Denmark freq	Norway freq	OR	*P* - value	*P* - het
DTNB	2p23.3	rs6746082	A	0.48	Broderik *et al.*	1.29	1.22×10^{-7}	0.76	0.76	0.77	0.75	1.19	1.62×10^{-10}	0.42
SP3	2q31.1	rs4325816	T	0.77	Went *et al.*	1.12	7.37×10^{-9}	0.78	0.77	0.77	0.76	1.08	5.16×10^{-3}	0.39
ULK4	3p22.1	rs1052501	C	0.19	Broderik *et al.*	1.32	7.47×10^{-9}	0.16	0.14	0.15	0.16	1.20	4.34×10^{-9}	0.26
ACTRT3, MYNN, LRRC34	3q26.2	rs10936599	C	0.76	Chubb *et al.*	1.26	8.7×10^{-14}	0.74	0.79	0.74	0.74	1.16	3.14×10^{-8}	0.65
ELL2	5q15	rs56219066	T	0.72	Swaminathan *et al.*	1.25	9.6×10^{-10}	0.74	0.71	0.75	0.74	1.15	3.80×10^{-8}	0.43
CEP120	5q23.2	rs6595443	T	0.45	Went *et al.*	1.11	1.20×10^{-8}	0.43	0.43	0.42	0.43	1.08	1.37×10^{-3}	0.85
JARID2	6p22.3	rs34229995	G	0.02	Mitchell *et al.*	1.37	1.31×10^{-8}	0.03	0.03	0.02	0.03	1.30	2.74×10^{-5}	0.57
PSORS1C2	6p21.3	rs2285803	T	0.26	Chubb *et al.*	1.19	9.67×10^{-11}	0.31	0.35	0.30	0.30	1.17	1.37×10^{-11}	0.13
ATG5	6q21	rs9372120	G	0.19	Mitchell *et al.*	1.18	9.09×10^{-15}	0.22	0.20	0.21	0.22	1.14	1.80×10^{-6}	0.88
CDCA7L	7p15.3	rs4487645	C	0.66	Broderik *et al.*	1.38	3.33×10^{-15}	0.66	1.00	0.67	0.66	1.26	5.20×10^{-15}	0.21
AC004917.1, CDC71L	7q22.3	rs17507636	C	0.74	Went *et al.*	1.12	9.20×10^{-9}	0.76	0.74	0.74	0.75	1.12	2.73×10^{-5}	0.25
POT1, RP11-3B12.1	7q31.33	rs58618031	T	0.73	Went *et al.*	1.12	2.73×10^{-8}	0.72	0.76	0.72	0.73	1.06	2.02×10^{-2}	0.68
SMARCD3	7q36.1	rs7781265	A	0.09	Mitchell *et al.*	1.19	9.79×10^{-9}	0.12	0.15	0.12	0.13	1.22	3.27×10^{-10}	0.81
CCAT1	8q24.21	rs1948915	C	0.33	Mitchell *et al.*	1.13	4.20×10^{-11}	0.37	0.34	0.36	0.34	1.09	2.20×10^{-4}	0.056
CDKN2A	9p21.3	rs2811710	C	0.64	Mitchell *et al.*	1.15	1.72×10^{-13}	0.66	0.62	0.65	0.65	1.10	2.19×10^{-5}	0.023
WAC	10p12.1	rs2790457	G	0.74	Mitchell *et al.*	1.12	1.77×10^{-8}	0.73	0.71	0.73	0.72	1.11	6.64×10^{-5}	0.15
CCND1	11q13.3	rs603965 (rs9344)	G	0.50	Weinhold *et al.*	1.82	2.92×10^{-10}	0.52	0.53	0.54	0.55	1.08	7.30×10^{-4}	0.29
PRR14, SRCAP, RP11-146F11.1	16p11.2	rs13338946	C	0.28	Went *et al.*	1.15	1.02×10^{-13}	0.27	0.27	0.27	0.25	1.12	6.39×10^{-6}	0.56
RFWD3	16q23.1	rs7193541	T	0.61	Mitchell *et al.*	1.13	5.00×10^{-12}	0.58	0.58	0.58	0.59	1.06	9.44×10^{-3}	0.65
TNFRSF13B	17p11.2	rs4273077	G	0.10	Chubb *et al.*	1.26	7.67×10^{-9}	0.11	0.08	0.10	0.10	1.32	3.33×10^{-14}	0.44
KLF2	19p13.11	rs11086029	T	0.23	Went *et al.*	1.14	6.79×10^{-11}	0.22	0.25	0.24	0.25	1.05	4.70×10^{-2}	0.88
PREX1	20q13.13	rs6066835	C	0.09	Mitchell *et al.*	1.26	1.36×10^{-13}	0.08	0.10	0.08	0.09	1.20	1.55×10^{-6}	0.024
HMGXB4, TOM1	22q13.1	rs138740	C	0.34	Swaminathan *et al*.	1.18	5.7×10^{-8}	0.37	0.42	0.37	0.38	1.08	6.37×10^{-4}	0.8
CBX7	22q13.1	rs877529	A	0.44	Chubb *et al.*	1.23	7.63×10^{-16}	0.46	0.45	0.44	0.47	1.15	1.12×10^{-9}	0.3

Broderick P *et al.* Common variation at 3p22.1 and 7p15.3 influences multiple myeloma risk. Nat Genet. 2012;44(1):58–61.
Chubb D *et al*. Common variation at 3q26.2, 6p21.33, 17p11.2 and 22q13.1 influences multiple myeloma risk. Nat Genet . 2013;45(10):1221–5.
Mitchell JS *et al.* Genome-wide association study identifies multiple susceptibility loci for multiple myeloma. Nat Commun. 2016;7(May).
Swaminathan B *et al.* Variants in ELL2 influencing immunoglobulin levels associate with multiple myeloma. Nat Commun. 2015;6(May):1–8.
Weinhold N *et al.* The CCND1 c.870G>A polymorphism is a risk factor for t(11;14)(q13;q32) multiple myeloma. Nat Genet. 2013;45(5):522–5.
Went M *et al.* Identification of multiple risk loci and regulatory mechanisms influencing susceptibility to multiple myeloma. Nat Commun. 2018;9(1):1–10.
