# Supplementary material for: Germline variants at SOHLH2 influence multiple myeloma risk
Source: Blood Cancer J. 2021 Apr 19;11(4):76. doi: 10.1038/s41408-021-00468-6 (PMC8055668; doi:10.1038/s41408-021-00468-6)
Supplement: Supplementary file 3 — Supplementary Table 2 [file 41408_2021_468_MOESM3_ESM.pdf]

Supplementary Table 2

Variants with 13q13 linkage disequilibrium block (r^2 > 0.8).

rsID	European MAF	Effect allele	Other allele	Sweden p-value	Iceland p-value	Denmark p-value	Norway p-value	combined p-value	combined odds ratio
rs200203825	4.47	AC	A	1.14E-03	1.10E-04	8.61E-05	4.16E-01	**2.65E-10**	1.38
rs145374408	3.54	C	CATA	1.28E-03	3.80E-04	6.06E-05	3.44E-01	**5.99E-10**	1.38
rs78351393	3.44	C	T	1.74E-03	3.75E-04	5.63E-05	3.42E-01	**7.61E-10**	1.38
rs76601148	3.45	G	A	1.80E-03	3.76E-04	5.64E-05	3.42E-01	**7.9E-10**	1.38
rs17202418	3.43	C	T	1.76E-03	3.70E-04	6.35E-05	3.50E-01	**8.57E-10**	1.38
rs74694612	3.44	T	C	2.61E-03	4.16E-04	4.31E-05	3.46E-01	**1.06E-09**	1.37
rs2149425	3.43	T	C	2.86E-03	3.77E-04	4.36E-05	3.48E-01	**1.08E-09**	1.37
rs2322895	3.43	T	C	2.89E-03	3.81E-04	4.35E-05	3.48E-01	**1.1E-09**	1.37
rs74656720	3.43	C	T	2.87E-03	3.81E-04	4.37E-05	3.48E-01	**1.1E-09**	1.37
rs78972162	3.43	C	T	2.88E-03	3.81E-04	4.41E-05	3.48E-01	**1.11E-09**	1.37
rs75933854	3.41	T	C	2.88E-03	3.93E-04	4.36E-05	3.48E-01	**1.14E-09**	1.37
rs77706449	3.43	T	C	2.89E-03	4.03E-04	4.36E-05	3.48E-01	**1.17E-09**	1.37
rs78518801	3.45	T	C	2.64E-03	3.78E-04	6.03E-05	3.90E-01	**1.45E-09**	1.37
rs75712673	3.45	G	T	2.79E-03	3.79E-04	6.05E-05	3.92E-01	**1.55E-09**	1.37
