# Supplementary material for: Germline variants at SOHLH2 influence multiple myeloma risk
Source: Blood Cancer J. 2021 Apr 19;11(4):76. doi: 10.1038/s41408-021-00468-6 (PMC8055668; doi:10.1038/s41408-021-00468-6)
Supplement: Supplementary file 4 — Supplementary Table 3 [file 41408_2021_468_MOESM4_ESM.pdf]

Supplementary Table 3

Predicted transcription factor binding motif changes for rs75712673, sorted by absolute log_2 fold change (FC).

Motif collection	motif	P-value T	P-value G	log2 FC
HOCOMOCO	FOXK1	2.75E-03	8.88E-06	8.2728
Swiss Regulon	IKZF2	2.38E-07	1.06E-04	-8.8005
JASPAR	EN1	8.98E-03	9.69E-05	6.5342
Swiss Regulon	SRY	2.30E-03	2.85E-05	6.3319
HT-SELEX	FOXJ3	2.06E-02	3.05E-04	6.0819
Swiss Regulon	EN1,2	6.41E-03	9.62E-05	6.0581
JASPAR	SRY	3.07E-03	5.15E-05	5.8967
HOCOMOCO	FOXD3	4.01E-03	8.03E-05	5.6416
HT-SELEX	FOXC2	5.02E-03	1.06E-04	5.5610
Swiss Regulon	SOX5	2.68E-03	6.10E-05	5.4591
Swiss Regulon	ZNF384	7.05E-05	2.93E-03	-5.3775
HOCOMOCO	FOXM1	3.39E-03	9.41E-05	5.1697
HT-SELEX	FOXG1	1.00E-03	3.13E-05	5.0012
HOCOMOCO	FOXO6	6.34E-03	2.01E-04	4.9813
HOCOMOCO	FOXP2	7.88E-03	2.94E-04	4.7431
HOMER	FOXO1	4.00E-03	1.60E-04	4.6453
HOCOMOCO	IRF7	2.80E-04	6.66E-03	-4.5724
HT-SELEX	FOXO1	1.04E-02	4.73E-04	4.4629
HOCOMOCO	FOXO3	1.00E-02	4.58E-04	4.4511
HT-SELEX	FOXJ3	5.58E-04	2.57E-05	4.4394
HOCOMOCO	FOXG1	5.65E-04	2.61E-05	4.4357
HOCOMOCO	SOX13	6.50E-03	3.05E-04	4.4128
HOCOMOCO	HOMEZ	2.39E-03	1.14E-04	4.3928
HOCOMOCO	BARX1	1.62E-03	8.17E-05	4.3109
JASPAR	STAT2+STAT1	7.13E-04	4.01E-05	4.1532
JASPAR	FOXO1	5.53E-03	3.11E-04	4.1525
HOMER	FOXA1	5.81E-03	3.60E-04	4.0133
Swiss Regulon	FOXP3	1.25E-06	2.02E-05	-4.0126
HT-SELEX	MYF6	7.41E-03	4.79E-04	3.9532
HOCOMOCO	SOX18	2.78E-03	1.80E-04	3.9478
HOCOMOCO	CPEB1	4.82E-04	7.43E-03	-3.9477
HOMER	FOXA1	3.93E-03	2.55E-04	3.9438
HOMER	TAL1	1.63E-03	1.06E-04	3.9414
HOCOMOCO	SOX5	5.03E-03	3.29E-04	3.9329
Swiss Regulon	HBP1_HMGB_SSRP1	3.65E-03	2.44E-04	3.9037
JASPAR	FOXP2	4.55E-03	3.10E-04	3.8769
Swiss Regulon	FOX{F1,F2,J1}	2.94E-03	2.01E-04	3.8716
HT-SELEX	FOXK1	4.78E-03	3.27E-04	3.8715
JASPAR	SOX5	1.73E-03	1.22E-04	3.8264
JASPAR	FOXF2	3.08E-03	2.32E-04	3.7326
Swiss Regulon	EVI1	8.75E-05	1.16E-03	-3.7311
Swiss Regulon	FOX{D1,D2}	4.09E-03	3.23E-04	3.6610
JASPAR	FOXA2	3.57E-03	2.82E-04	3.6608

Swiss Regulon	FOXA2	3.57E-03	2.82E-04	3.6608
HT-SELEX	FOXB1	6.10E-03	4.83E-04	3.6602
HOCOMOCO	PRDM6	6.63E-06	8.19E-05	-3.6285
HOCOMOCO	FOXF1	4.21E-03	3.42E-04	3.6226
HT-SELEX	FOXK1	2.80E-03	2.32E-04	3.5912
HOMER	SPDEF	5.02E-03	4.17E-04	3.5909
Swiss Regulon	ZBTB16	6.53E-05	7.86E-04	-3.5900
HOCOMOCO	FOXC1	4.48E-03	3.87E-04	3.5314
HOCOMOCO	NFAT5	1.10E-04	1.25E-03	-3.5029
HOCOMOCO	FOXF2	1.90E-03	1.69E-04	3.4937
HOCOMOCO	FOXJ3	3.01E-03	2.73E-04	3.4597
JASPAR	NFATC2	8.63E-05	9.46E-04	-3.4534
Swiss Regulon	FOXO1,3,4	2.66E-03	2.44E-04	3.4491
Swiss Regulon	ETS1,2	1.99E-03	1.91E-04	3.3792
HOCOMOCO	FOXQ1	4.50E-03	4.37E-04	3.3641
HT-SELEX	FOXG1	1.41E-03	1.43E-04	3.3090
HOCOMOCO	SOX11	4.43E-03	4.58E-04	3.2730
HT-SELEX	SOX3	3.24E-03	3.60E-04	3.1676
HT-SELEX	SOX2	3.24E-03	3.78E-04	3.0971
HOMER	FLI1	1.89E-03	2.25E-04	3.0679
HT-SELEX	E2F2	5.32E-04	6.53E-05	3.0275
HT-SELEX	FOXO3	1.04E-03	1.29E-04	3.0127
HT-SELEX	SOX2	3.75E-03	4.83E-04	2.9565
HT-SELEX	SOX2	2.80E-03	3.97E-04	2.8163
HT-SELEX	E2F2	1.56E-03	2.21E-04	2.8154
HOCOMOCO	ZN384	1.17E-04	7.92E-04	-2.7585
Swiss Regulon	FOXQ1	2.80E-03	4.17E-04	2.7458
HOMER	ERG	7.47E-04	1.12E-04	2.7426
HT-SELEX	SOX15	2.16E-03	3.26E-04	2.7275
HOCOMOCO	SOX21	1.11E-03	1.71E-04	2.6988
HT-SELEX	FOXC1	1.72E-03	2.82E-04	2.6068
HOCOMOCO	ZN563	1.00E-05	1.81E-06	2.4691
HT-SELEX	ETV6	2.19E-03	3.97E-04	2.4640
HOCOMOCO	NFAC2	3.21E-04	1.70E-03	-2.4047
HT-SELEX	ETV2	5.59E-04	1.06E-04	2.3936
HT-SELEX	SPDEF	2.82E-04	1.48E-03	-2.3933
JASPAR	FOXP1	1.16E-03	2.32E-04	2.3232
HOCOMOCO	SOX7	2.04E-03	4.19E-04	2.2825
Swiss Regulon	PAX4	1.91E-04	9.10E-04	-2.2527
HOCOMOCO	NFAC4	1.48E-04	6.42E-04	-2.1206

TFBS:transcription factor binding site, SNP: single nucleotide polymorphism.
